# Supplementary material for: The Disease Gene THAP12 is a Transcriptional Regulator of Mitochondrial ETC Complex I
Source: bioRxiv. 2026 Jul 17:2026.07.16.738975. Preprint. [Version 1] doi: 10.64898/2026.07.16.738975 (PMC13404843; doi:10.64898/2026.07.16.738975)
Supplement: Supplement 4 [file NIHPP2026.07.16.738975v1-supplement-4.pdf]

bioRxiv preprint doi: https://doi.org/10.64898/2026.07.16.738975; this version posted July 17, 2026. The copyright holder for this preprint (which was not certified by peer review) is the author/funder, who has granted bioRxiv a license to display the preprint in perpetuity. It is made available under a CC-BY 4.0 International license.

SUPPLEMENTAL FIGURES

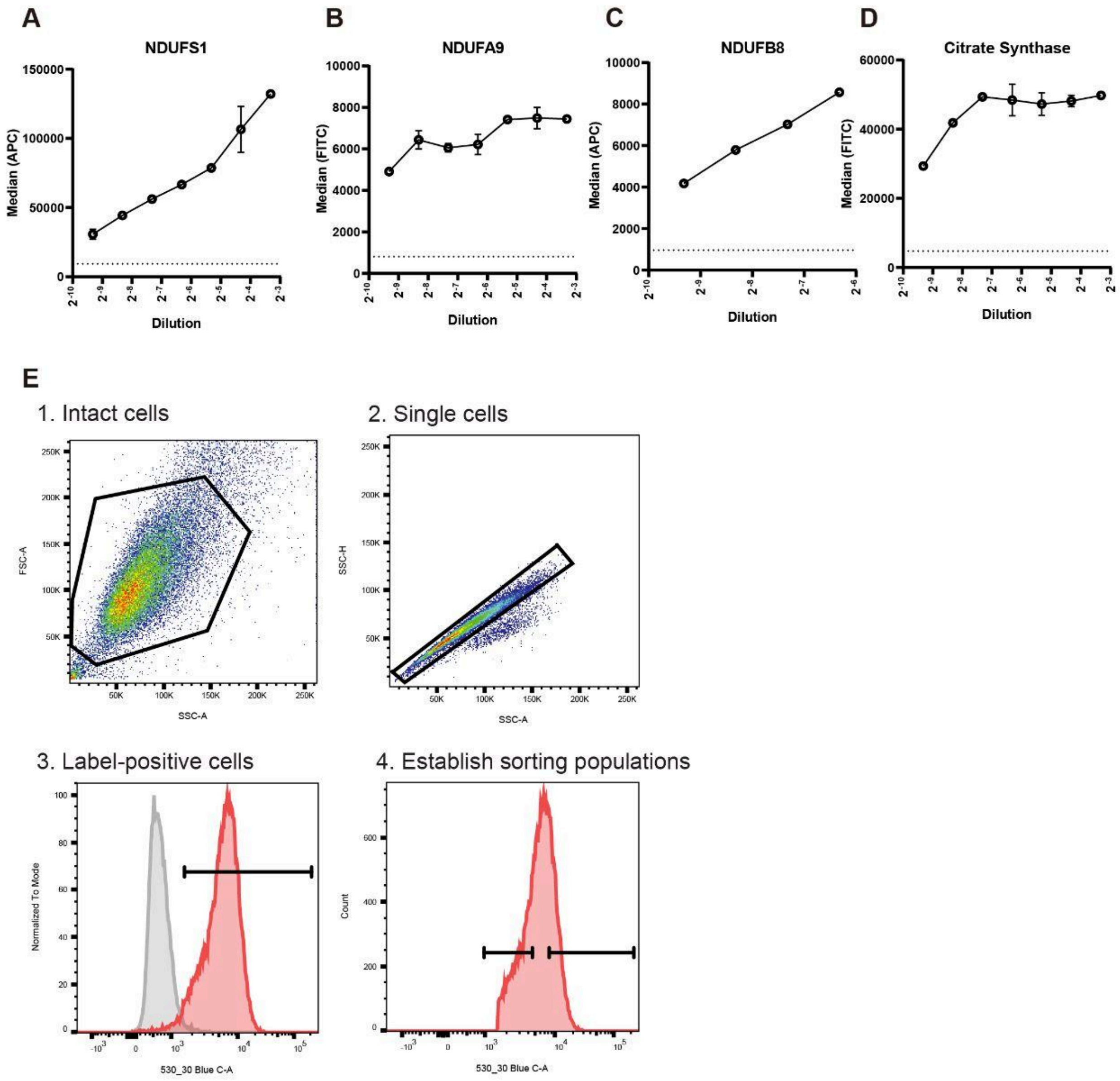

Supplemental Figure 1. Optimization of primary antibody staining and depiction of gating strategy for FACS CRISPR screen Primary antibody titration curves depicted for antibodies used in screens. Antibodies include NDUFS1 (A), NDUFA9 (B), NDUFB8 (C, and Citrate Synthase (D). (B) Depiction of gating strategy used for FACS screen. Samples were first gated for intact (1) and singularized (2) cells based on their forward- and side light scattering. Singularized cells are assessed for fluorescence signal by comparing against a secondary antibody-only negative control (3). Cells with true positive fluorescence were then gated based on their relative fluorescence signal. Cells within the bottom 30% or top 30% for fluorescence signal were collected for barcode sequencing.

bioRxiv preprint doi: https://doi.org/10.64898/2026.07.16.738975; this version posted July 17, 2026. The copyright holder for this preprint (which was not certified by peer review) is the author/funder, who has granted bioRxiv a license to display the preprint in perpetuity. It is made available under a CC-BY 4.0 International license.

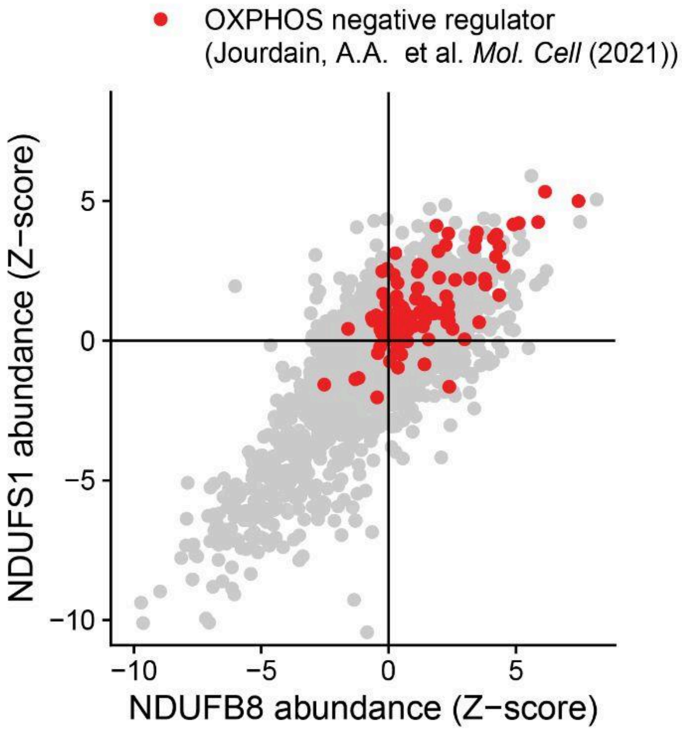

Supplemental Figure 2. Comparison of Complex I screen results to galactose viability screen. NDUFS1 and NDUFB8 CRISPR screen compared against each. Top t0 hits from a previously performed galactose are denoted in red.

bioRxiv preprint doi: https://doi.org/10.64898/2026.07.16.738975; this version posted July 17, 2026. The copyright holder for this preprint (which was not certified by peer review) is the author/funder, who has granted bioRxiv a license to display the preprint in perpetuity. It is made available under a CC-BY 4.0 International license.

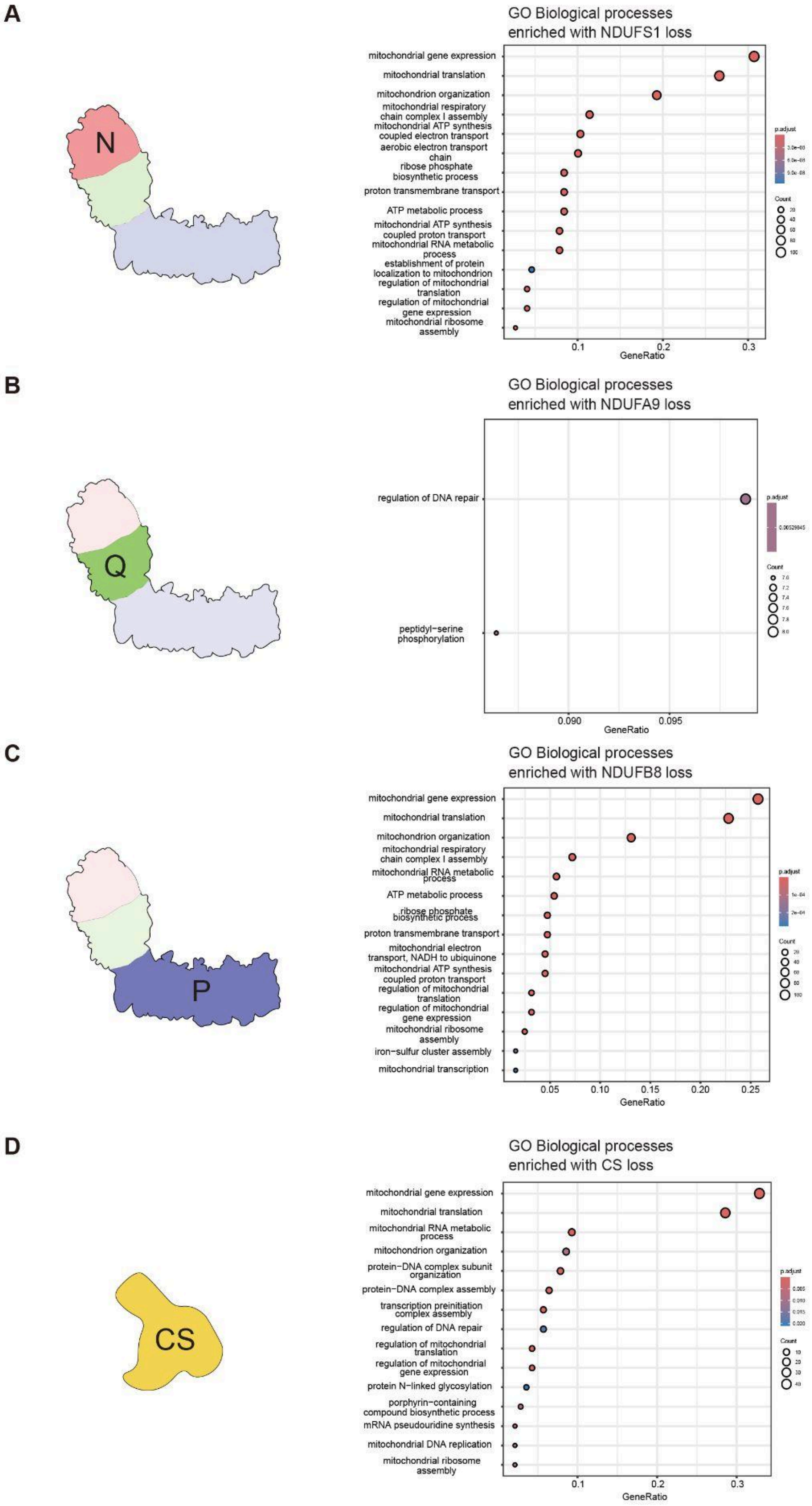

bioRxiv preprint doi: https://doi.org/10.64898/2026.07.16.738975; this version posted July 17, 2026. The copyright holder for this preprint (which was not certified by peer review) is the author/funder, who has granted bioRxiv a license to display the preprint in perpetuity. It is made available under a CC-BY 4.0 International license.

Supplemental Figure 3. Summary of biological processes associated with Complex I loss across CRISPR screens. Gene ontology assessment for biological processes represented on the depleting side of the screens. Biological processes are depicted for the NDUFS1 (A), NDUFA9 (B), NDUFB8 (C, and Citrate Synthase (D) screens.

bioRxiv preprint doi: https://doi.org/10.64898/2026.07.16.738975; this version posted July 17, 2026. The copyright holder for this preprint (which was not certified by peer review) is the author/funder, who has granted bioRxiv a license to display the preprint in perpetuity. It is made available under a CC-BY 4.0 International license.

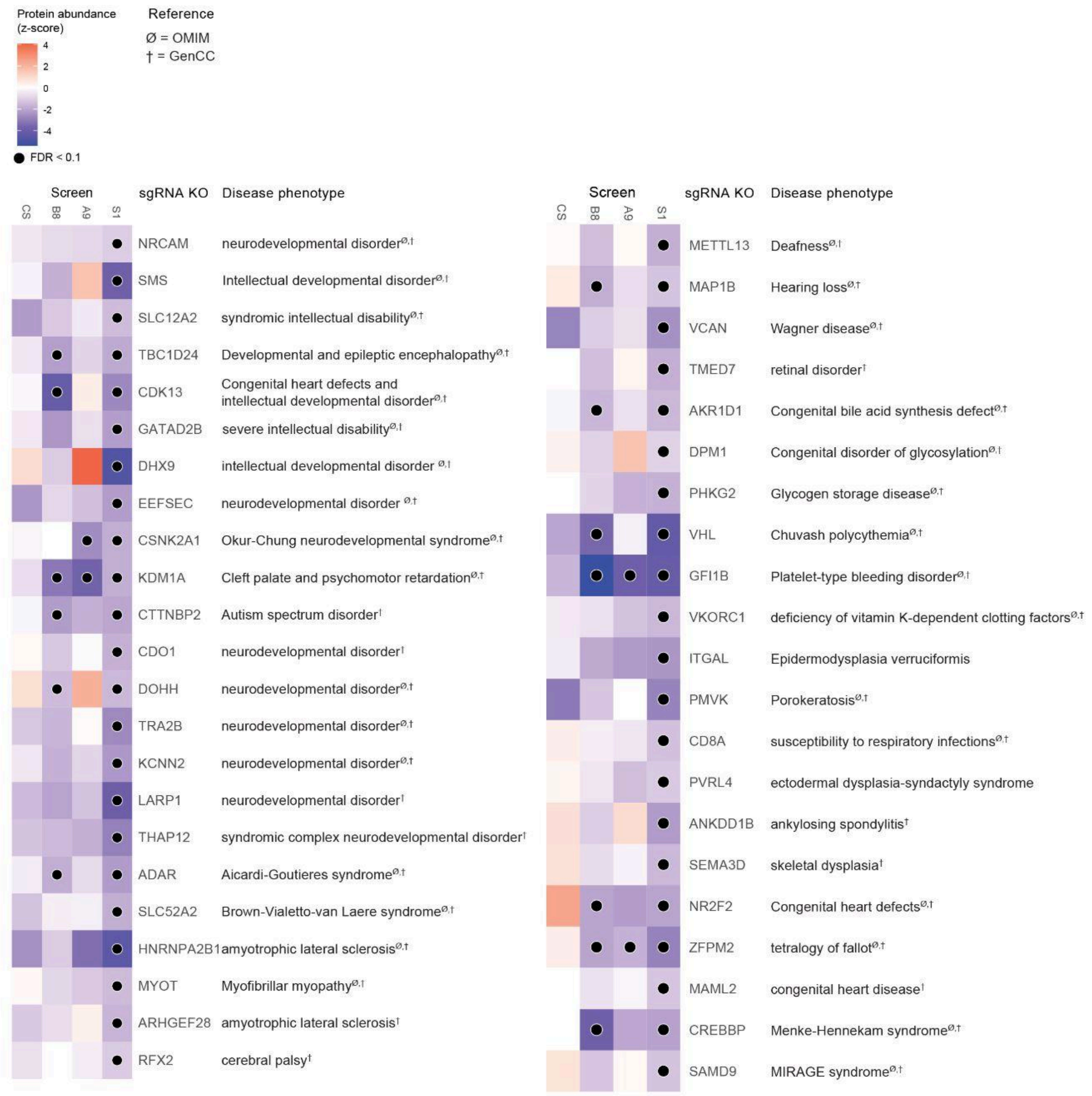

Supplemental Figure 4. Human disease phenotypes associated with non-mitochondrial modifiers of Complex I. Non-mitochondrial genes, as evaluated by their presence in MitoCarta 3.0, were filtered based on the presence of a human disease phenotype. Human disease phenotypes were identified based on their presence in Online Mendelian Inheritance in Man (OMIM) and Gene Curation Coalition (GenCC) databases.

bioRxiv preprint doi: https://doi.org/10.64898/2026.07.16.738975; this version posted July 17, 2026. The copyright holder for this preprint (which was not certified by peer review) is the author/funder, who has granted bioRxiv a license to display the preprint in perpetuity. It is made available under a CC-BY 4.0 International license.

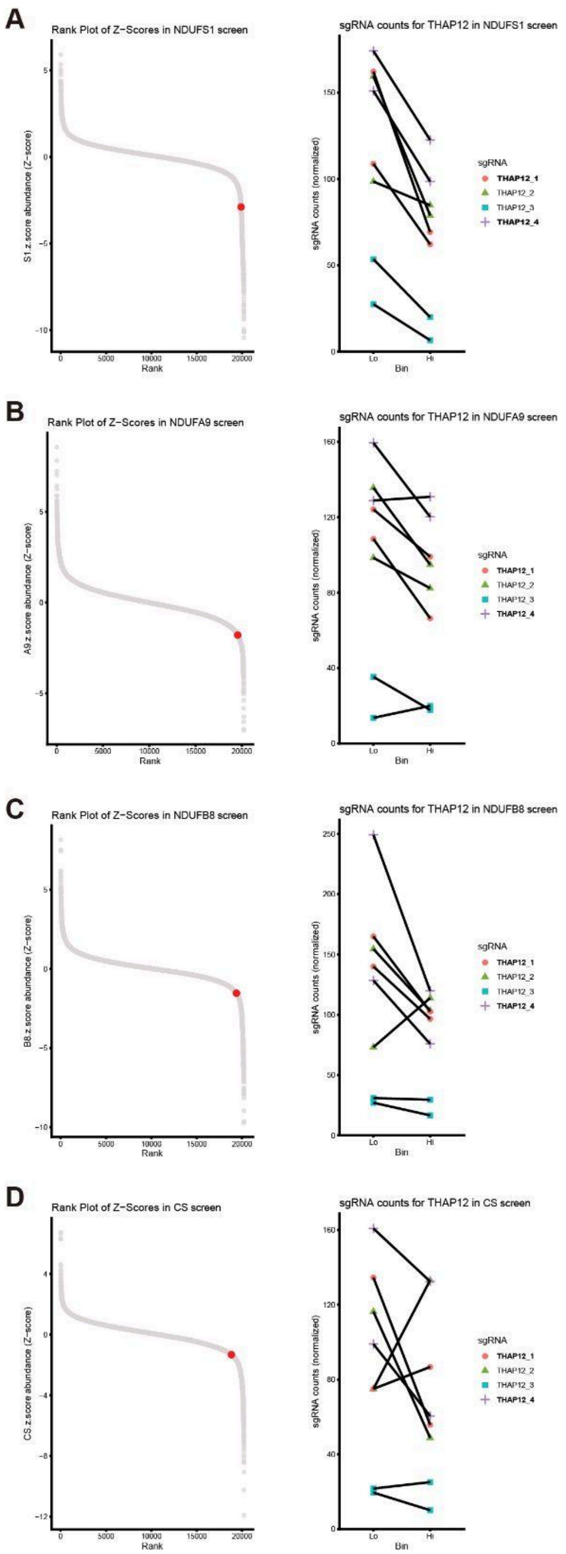

Supplemental Figure 5. THAP12 results by screen. THAP12 results from the NDUFS1 (A), NDUFA9 (B), NDUFB8 (C), and Citrate Synthase (D) screens. For each screen, THAP12 is highlighted in the rank plot (left) alongside its per-guide results (right).

bioRxiv preprint doi: https://doi.org/10.64898/2026.07.16.738975; this version posted July 17, 2026. The copyright holder for this preprint (which was not certified by peer review) is the author/funder, who has granted bioRxiv a license to display the preprint in perpetuity. It is made available under a CC-BY 4.0 International license.

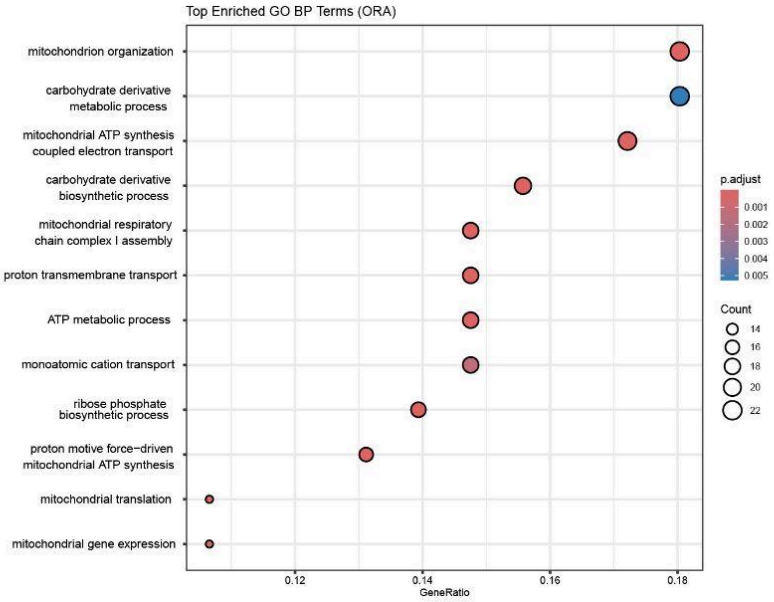

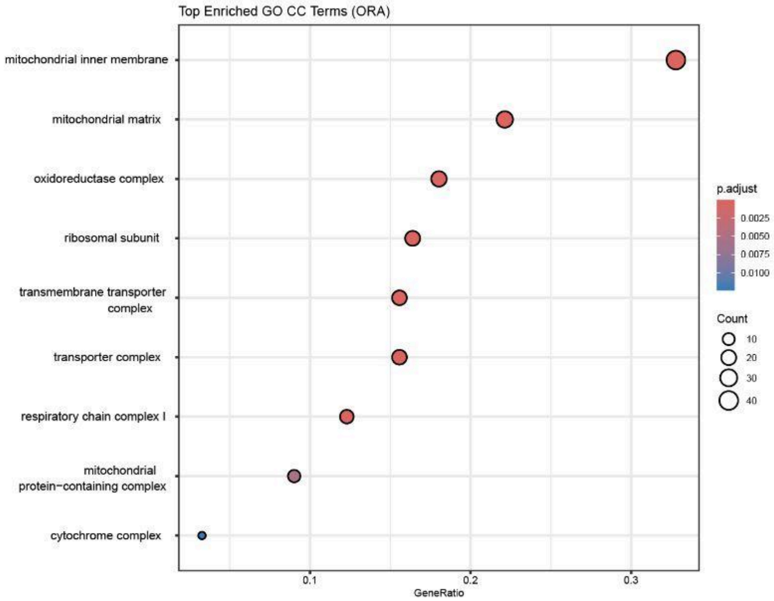

Supplemental Figure 6. Disrupted processes identified in THAP12 KO proteomics. Gene ontology analysis of proteomics data from THAP12 KO K562 cells depicting the biological processes (top) and cellular compartments (bottom) associated with the most depleted proteins.

bioRxiv preprint doi: https://doi.org/10.64898/2026.07.16.738975; this version posted July 17, 2026. The copyright holder for this preprint (which was not certified by peer review) is the author/funder, who has granted bioRxiv a license to display the preprint in perpetuity. It is made available under a CC-BY 4.0 International license.

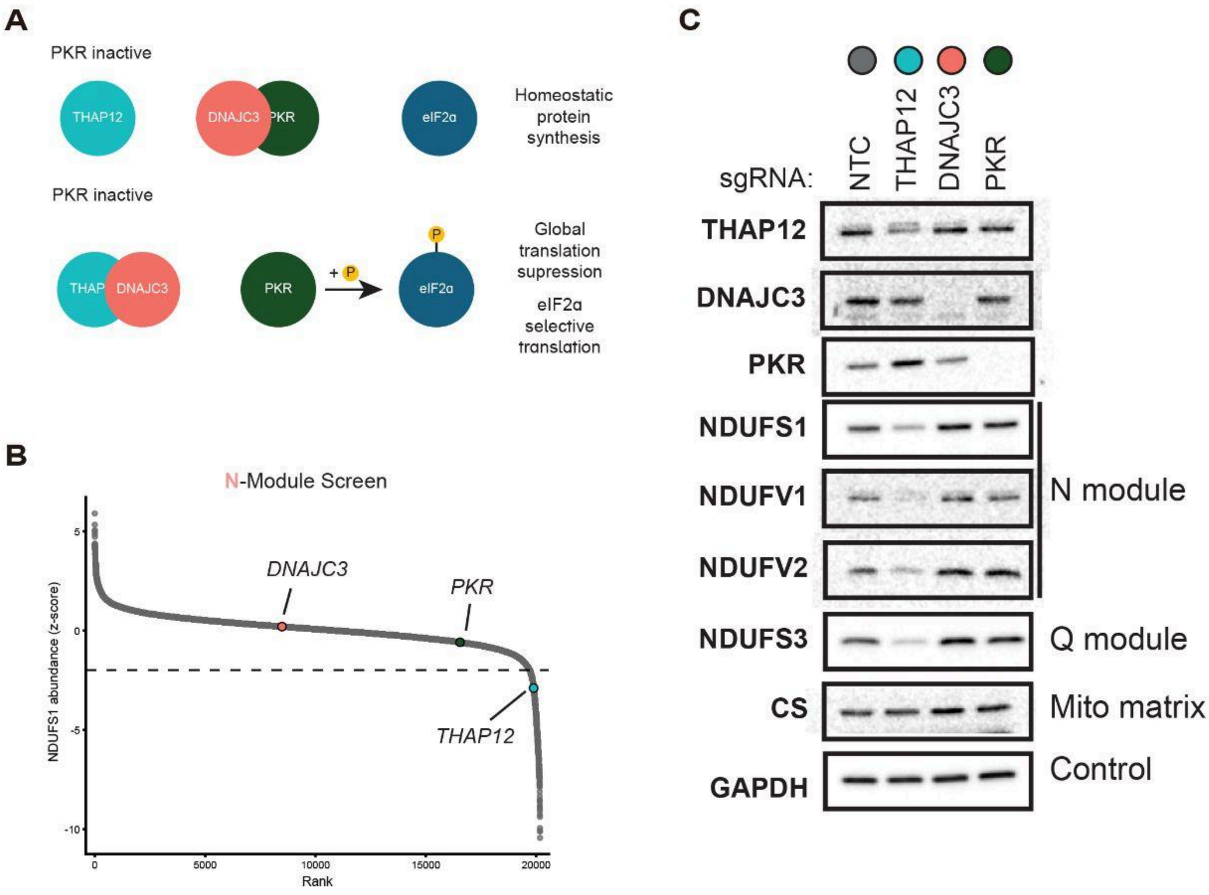

Supplemental Figure 7. Disrupted PKR signaling does not explain CI deficiency with THAP12 loss. (A) Previously proposed model for THAP12 in regulating PKR signaling through direct interaction with DNAJC3. (B) Evaluation of results for PKR signaling members in the NDUFS1 N module screen. (C) Assessment of CI protein abundance with sgRNA KOs of individual members of the PKR signaling cascade.

bioRxiv preprint doi: https://doi.org/10.64898/2026.07.16.738975; this version posted July 17, 2026. The copyright holder for this preprint (which was not certified by peer review) is the author/funder, who has granted bioRxiv a license to display the preprint in perpetuity. It is made available under a CC-BY 4.0 International license.

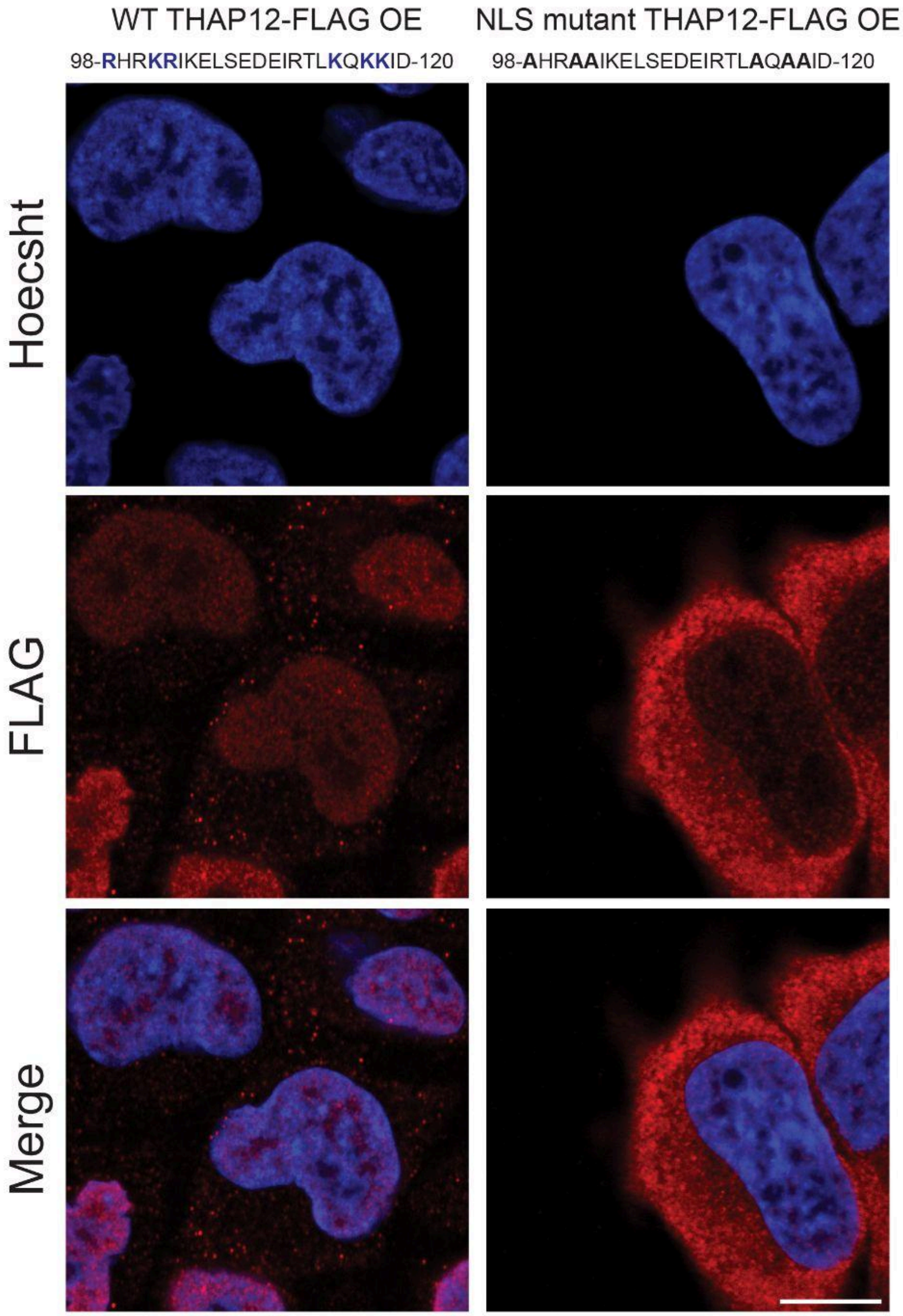

Supplemental Figure 8. THAP12 nuclear localization is largely coordinated by a bipartite nuclear localization signal (NLS). Assessment of THAP12 localization after neutralizing a predicted NLS sequence. Basic residues imparting a positive charge for nuclear localization are depicted in blue (left). These basic residues were converted to alanine to neutralize the positive charge. These changes are depicted with bold, black lettering (right).

bioRxiv preprint doi: https://doi.org/10.64898/2026.07.16.738975; this version posted July 17, 2026. The copyright holder for this preprint (which was not certified by peer review) is the author/funder, who has granted bioRxiv a license to display the preprint in perpetuity. It is made available under a CC-BY 4.0 International license.

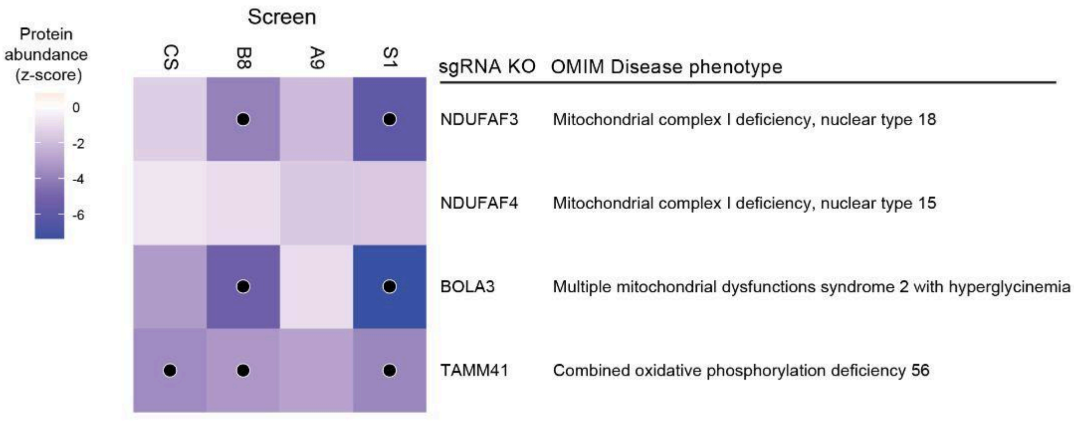

Supplemental Figure 9. THAP12 controls a transcriptional node required for CI biogenesis and implicated in mitochondrial disease. Results from CI CRISPR screens for defined THAP12 gene targets. Cells are labeled with a dot to denote an FDR < 0.1. THAP12 targets are additionally annotated with their respective disease phenotype annotation present in OMIM.

bioRxiv preprint doi: https://doi.org/10.64898/2026.07.16.738975; this version posted July 17, 2026. The copyright holder for this preprint (which was not certified by peer review) is the author/funder, who has granted bioRxiv a license to display the preprint in perpetuity. It is made available under a CC-BY 4.0 International license.

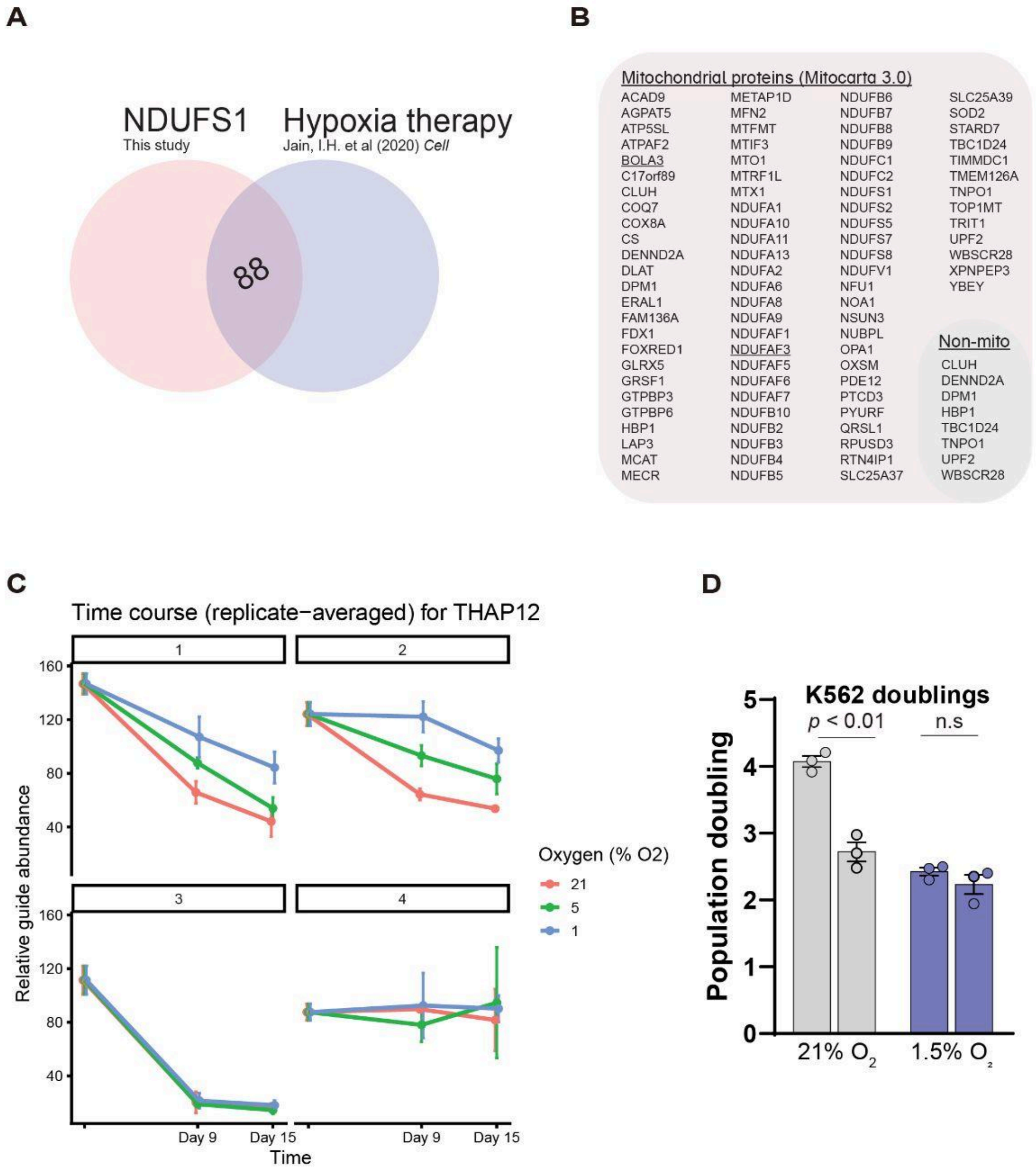

Supplemental Figure 10. THAP12 disease as a candidate for hypoxia therapy. (A) Comparison of screen results from the NDUFS1 screen (this work) and the previously performed hypoxia therapy screen (B) List of genes identified in screen comparison analysis that are further grouped based on mitochondrial localization. (C) Guide-level analysis for THAP12 sgRNA KO cells in the hypoxia therapy screen, relevant to Fig. 6E. (D) Validation of hypoxia therapy result using monoclonal THAP12 KO K562 cells. This data is a non-normalized representation of data presented in Fig. 6F
